# Methodology for developing quality indicators for the care of older people in the Emergency Department

**DOI:** 10.1186/1471-227X-13-23

**Published:** 2013-12-06

**Authors:** Melinda Martin-Khan, Ellen Burkett, Linda Schnitker, Richard N Jones, Leonard C Gray

**Affiliations:** 1 Centre for Research in Geriatric Medicine, Level 2, Building 33, Princess Alexandra Hospital, The University of Queensland, Ipswich Road, Woolloongabba QLD 4102, Australia; 2 Centre for Online Health, Level 3, Foundation Building, royal Children’s Hospital, The University of Queensland, Herston QLD 4029, Australia; 3 Emergency Department, Princess Alexandra Hospital, Queensland Health, Ipswich Road, Woolloongabba QLD 4102, Australia; 4 Hebrew SeniorLife, Institute for Aging Research, Beth Israel Deaconess Medical Centre, Harvard Medical School, Cambridge, 1200 Centre Street, Boston, MA 02131, USA

**Keywords:** Emergency service, Hospital, Quality indicators, Health care, Geriatrics, Health services for the aged, Dementia, Cognitive impairment, Residential facilities, Protocol

## Abstract

**Background:**

Compared with younger people, older people have a higher risk of adverse health outcomes when presenting to emergency departments. As the population ages, older people will make up an increasing proportion of the emergency department population. Therefore it is timely that consideration be given to the quality of care received by older persons in emergency departments, and to consideration of those older people with special needs. Particular attention will be focused on important groups of older people, such as patients with cognitive impairment, residents of long term care and patients with palliative care needs. This project will develop a suite of quality indicators focused on the care of older persons in the emergency department.

**Methods/design:**

Following input from an expert panel, an initial set of structural, process, and outcome indicators will be developed based on thorough systematic search in the scientific literature. All initial indicators will be tested in eight emergency departments for their validity and feasibility. Results of the data from the field studies will be presented to the expert panel at a second meeting. A suite of Quality Indicators for the older emergency department population will be finalised following a formal voting process.

**Discussion:**

The predicted burgeoning in the number of older persons presenting to emergency departments combined with the recognised quality deficiencies in emergency department care delivery to this population, highlight the need for a quality framework for the care of older persons in emergency departments. Additionally, high quality of care is associated with improved survival & health outcomes of elderly patients. The development of well-selected, validated and economical quality indicators will allow appropriate targeting of resources (financial, education or quality management) to improve quality in areas with maximum potential for improvement.

## Background

Currently older persons make up an important group of patients served by Emergency Departments (EDs). The elderly have higher rates of utilisation of emergency services than other patient groups; in developed countries, older people represent 12% to 21% of all ED encounters [1]. The proportion of older people aged 60 years and over is expected to rise from 19% in 2000 to 34% by 2050 [2], resulting in a commensurate increase in ED presentations by older persons. Awareness of the connection between ED use and the health of older people, has led to an increased focus on the quality of geriatric emergency medical care and patient outcomes [3-5].

Emergency practice is characterised by high volumes of high acuity and high complexity patients. This, combined with often-incomplete information and frequent interruptions, creates an environment prone to error [6,7]. Older people have been identified as a particularly vulnerable population in ED, having substantially inferior clinical outcomes, with higher rates of missed diagnoses, and medication errors, when compared with younger, severity-matched controls [8-12]. Older persons discharged from ED are at high risk of adverse outcomes, such as functional decline, ED re-admission and hospitalisation, death, and institutionalisation [12-17].

While the quality of care for older people is a key issue, there may also be a need to consider older people with special needs as a separate sub-group as they may have some additional significant quality of care issues. There is evidence that older ED patients with cognitive impairment, which is common in the older ED population [18-20], have an increased risk of bad outcomes and events [17,21,22]. Along with issues associated with being older, older persons with cognitive impairment, who may experience problems with their memory, reasoning, insight, or their ability to learn, have special needs when presenting to busy ED environments. Another second significant sub-group includes people residing in long term care. Persons living in long term care are in general older, have complex medical histories and are more likely to present to the ED with cognitive impairment [23]. They experience longer waiting hours, are resource intensive, are more likely to die in hospital [24,25]. A third important sub-group includes older people at the end-of-life. The chaotic ED environment can be particularly burdensome for older patients requiring palliative care. A study by Beyon *et al*. found that among older people who died in ED, over half of them presented to the ED with a diagnosis that triggered palliative care [26]. However, in ED palliative care is often not provided [27].

High quality care has been shown to be associated with improved survival and health outcomes of elderly patients [28]. The anticipated “greying” of the population, with its attendant increase in older ED patient attendances, mandates an evaluation of the capacity of EDs to deliver quality care to this vulnerable patient group. Accurate assessment of current levels of quality of care in EDs is required to enable a targeted approach to care that is identified as inadequate, to improve patient outcomes. Quality indicators allow levels of performance to be determined and, as part of a quality management system, provide opportunity for benchmarking and improved care delivery [29]. The development of a comprehensive set of quality indicators (QIs) will aid in improving delivery of care in the ED to the geriatric population. This will be timely in the context of the anticipated burgeoning in the numbers of elderly presenting to EDs. In order to be considered valid, QIs should be [29,30]:

1. Specific & defined, with content validity in the QI definition (including a defined numerator, denominator, clinical exclusions to the denominator & covariates used for risk adjustment)

2. Meaningful with evidence to link them to the desired outcome

3. Structured to facilitate comparison of care delivery between facilities

4. Amenable to improvement by each particular facility, and

5. Efficiently measurable.

Review of the literature revealed one previous publication of a group of ED-specific QIs aimed at geriatric patients [31]. These, proposed by the Society for Academic Emergency Medicine (SAEM) indicators, pertain to 3 clinical domains (cognitive assessment, pain, and transitional care) and have a predominant focus on process of care, rather than structure or outcome. The data for the process indicators are derived from chart audit, but no field testing data is available in the scientific literature. After creating scoring rules, Schnitker *et al*. used the SAEM QIs for cognitive assessment, in a geriatric ED population (N = 277) and found that cognitive assessment and its documentation in medical records occurred in too few patients such that scoring the majority of the QIs was impracticable in this sample [32].

The aim of this project is to determine predictors of quality of care of geriatric patients in EDs, and to develop a suite of QIs, including structural, process and outcome measures, that are feasible with minimal collection cost, whilst being reflective of true levels of quality delivered, for use in ED-care of the elderly. This will include the potential to propose a sub-set of QIs focused on the special needs of 1) older ED patients with cognitive impairment 2) those residing in nursing homes presenting to EDs, 3) and older ED patients with palliative care needs.

## Methods/design

**Figure 1:**
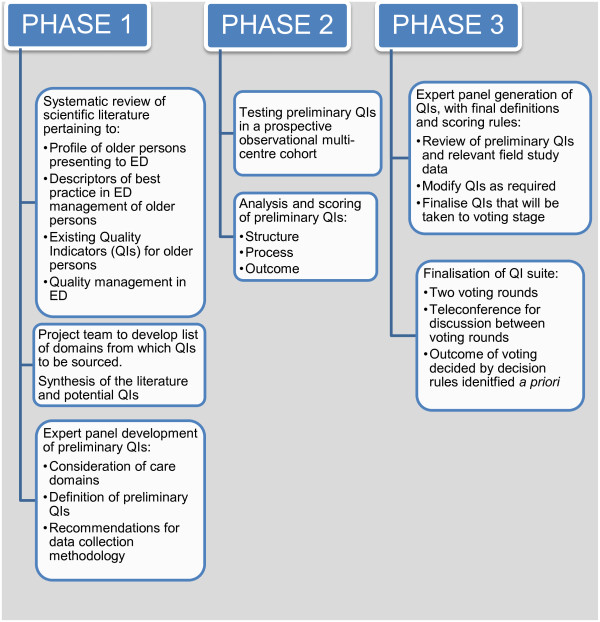
Schematic of the study design.

To ensure that a suite of quality indicators for the care of older persons in the ED is developed using an evidence-based approach that reflects the diversity of ED systems in developed nations, a three-phase mixed methods study was designed (Figure 1). The project will consist of: 1) a review of the scientific literature and expert panel input for the development of a preliminary suite of indicators; 2) field study of preliminary indicators at 8 Australian emergency services; 3) a facilitated panel discussion among key experts in emergency and geriatric medicine followed by a formal voting process, resulting in a final QI suite. The results of each phase will inform subsequent phases.

### Ethics

Research ethics board approval was received for the project from Metro South Human Research Ethics Committee (HREC/11/QPAH/628); Australian Capital Territory (ACT) Government Health Human Research Ethics Committee Low Risk Sub-committee (ETHLR.12.097); The University of Queensland Behavioural & Social Sciences Ethical Review Committee (2012000631); and Melbourne Health Human Research Ethics Committee (2012.010). Site Specific Governance approval was received for this project from Metro South Centres for Health Research Governance (SSA/11/QPAH/628; SSA/12/QPAH/211); Metro North Health Service District Research, Ethics and Governance Unit (SSA/12/QPCH/76); West Moreton Health Service District Human Research Ethics Committee & Research Governance Office (SSA/12/QWMS/23); and Northern Health Research Governance Office (SSA/12/NH/4).

For the field study, research nurses will obtain informed written consent from participating patients at each site.

## Phase 1: Review of the literature

### Objective

The purpose of this phase is to develop a preliminary QI set through a process of evaluation of available scientific literature, analysis of data collected from a pilot study [32], and finally, expert panel input. There will be a focus on utilising structural, process and outcome measures. Specific areas of interest include: triage, clinical assessment, cognition and cognitive assessment, delirium, palliative care, medication and other geriatric specific syndromes; the expert panel will be able to nominate additional topic areas believed to be of high priority.

#### *Expert panel*

A range of stakeholders will be sought to establish the ED expert panel. The study team developed a list of stakeholder categories to identify the range of expertise required, such as physicians, nurses, dementia specialists or QI development experts (Table 1). In the first instance, one representative from each data collection site (field study) will be invited to participate in the panel. Purposeful sampling will follow, to populate each category with at least one representative. The total panel will include 12–18 participants. Potential participants will be contacted by email with an explanation of the study and an invitation to join the expert panel. Panel members will be required to participate in two face-to-face expert panel meetings and a formal voting process, which will be conducted after the second panel meeting. Final Distribution of panel members is noted in Table 1.

**Table 1:** Expert panel members

**Participant categories**	**Number on panel**
Allied Health representative	1
Consumer representative	1
Emergency department pharmacist	1
Emergency medicine nurse	3
Emergency medicine specialist	7
Geriatric medicine specialist	4
Geriatric nurse	1
Quality indicator/improvement expert	1^*^

*Many people on the panel crossed over more than one category, but for the purpose of this table each panel member was only represented once.

### Design

The scientific literature will be evaluated systematically to address 4 core concept areas:

1. Profile of elderly patients presenting to EDs including: patient characteristics; presenting complaints; discharge diagnoses; discharge destinations; predictors of failed discharge from ED in elderly; predictors of morbidity & mortality within 28 days subsequent to ED discharge of elders

2. Descriptors of best practice in assessment and management of geriatric ED patients, in terms of process, environment and structure including strength of relationship of each to desired outcomes

3. Existing QIs for elderly patients in ED and, where relevant, non-ED settings

4. Quality management in ED including: structure and feasibility of QIs; barriers to achieving quality of care in EDs; benchmarking in EDs; quality improvement projects in EDs.

National Health and Medical Research Council (NHMRC) guidelines for systematic review of scientific literature will be followed for each core concept [33]. This will include the identification of relevant MeSH/search terms; a search of the peer-reviewed and gray literature; and a hand search of bibliography and reference lists. Using the identified literature, a preliminary list of potential domains for sourcing QIs will be formulated (EB, LS). The resultant literature summary and the preliminary list of potential QI-domains will then be distributed to an expert panel for review, and to initiate discussion at the expert panel meeting.

The first time, the expert panel will meet for two days. The meeting will commence with a presentation of the study, an overview of QI development methodology and a discussion of potential data collection tools. For the remaining time, the Chair (MMK) will lead the panel through a formal process of review for each domain. This will include: a general discussion of the literature; review of existing QIs (if any) with suggestions for modification if required; consideration of new potential QIs based on the study team’s review of the literature [12,34]; and opportunity for the panel to recommend new QIs. The resultant preliminary indicators will aim to encompass assessment of emergency department structure (including the physical environment and the policies related to the care of older persons), process and outcomes.

### Data collection

Throughout the meeting, three scribes will record decisions and concepts resulting from the discussion; and each panel member will informally rate potential QIs based on three criteria, including validity, significance, and responsibility. These ratings will be recorded on individual data collection sheets. This will be used as an additional resource to ensure that the scribes captured all relevant discussion points.

### Data compilation

After completion of the first expert panel meeting, three investigators (EB, LS, MMK) will review all the preliminary indicators. A working manual for each indicator set will be established (structural, process, outcome). Each preliminary QI will be defined - this includes detailed specification of the numerator, denominator, exclusion characteristics and any factors that will be significant for risk adjustment. The feedback from the expert panel will be incorporated into the manual alongside each indicator. Any preliminary indicators rejected at the expert panel meeting as clearly unsuitable will be recorded, along with the justification for exclusion, in a separate manual, known as The Excluded Indicators Manual.

## Phase 2: Field study

### Objective

The purpose of the field study is to test the feasibility and usefulness of each of the preliminary indicators suggested in Phase 1. This will be achieved by collecting data from a representative sample of older patients presenting for emergency department care. The assessment of potential QIs will include a complex analytic process that involves risk adjustment.

### Design

The study will be a multi-centre prospective observational cohort study of the validity and feasibility of the preliminary QIs developed by the study team, including any previously published relevant QIs [35].

Working from the defined preliminary indicators recorded in each of the working manuals, a matrix of data items and data collection methods will be created which ensures that, for each potential QI, the relevant data items have been identified and a collection method found (EB, LS, MMK). Based on the data matrix, the data collection tools will be designed. Wherever possible, existing, validated, tools will be used for the data collection. If a tool cannot be identified to collect specific data, then a data collection tool will be designed. The tool will be tested for feasibility at several sites at the beginning of the project and feedback from the research nurses will enable refinement of the tool.

If feasible, data to score all QIs will be collected. In addition, if feasible, additional data to support the internal validity of the QI will also be collected. It is anticipated that data collection methods will include:

1. Site visit

2. Direct patient assessment

3. Review of the patient chart

4. Extraction of data from the electronic information system in ED

5. Phone follow-up at consecutive time intervals (7 and 28 days) with patient

6. State held ED and hospital episode data.

QIs will only be excluded at this point if a novel data collection method is identified (separate from the above list), and the cost of additional data collection is prohibitive. Any indicators excluded at this point will be recorded, with the justification, in the Excluded Indicators Manual.

#### Sample size

The sample size is determined in two ways using simulation methods resulting in a required sample of 480 participants. This planned sample size will have 77% power to detect reliability coefficients within an acceptable level of precision (estimated correlation among raters coefficient greater than 0.35 when the true value is 0.6 and the QI base rate is 50%). Given these parameters, for the classification analysis, we will be able to correctly classify units as poor (proportion of patients flagging a QI is less than the observed 20th percentile across facilities and the true quality score for the facility is below the 20^th^ percentile) with an overall 83% accuracy. The empirical c-statistic for this classification (proportion of facilities with true performance in the lowest 20% that have observed quality scores in the lowest 20%) is 0.98.

#### *Participants or study groups*

The minimum number required from each site will be 60 cases. We will aim to recruit 80 cases across eight units to allow for incomplete data. This will enable a final sample size of at least 480 cases. Site selection will be influenced by case-mix and ability to recruit adequate patient numbers, with the final group allowing for representation of district, metropolitan and tertiary Emergency Departments.

#### *Inclusion criteria*

All patients aged 70 years and older presenting to study site emergency departments during the study period will be considered eligible for enrolment.

#### *Exclusion criteria*

1. Patients who have presented to the ED and have completed triage 2 or more hours prior to the Research Nurse being available to approach them for consent

2. Patients presenting to ED with acute illness of such severity that prevents staff from gaining consent (either from the individual or their caregiver)

3. Patients returning to the ED, after already being consented for participation at the initial ED visit. Aspects relevant to their return to ED will be identified via the phone follow up process, from chart audit and from State held data on ED visits

4. For non-English speaking patients, staff will attempt to identify a suitable interpreter to seek consent for participation – if no interpreter can be found in a suitable time-frame then the patient will be excluded

5. Patients need to be able to participate in the planned phone follow-up – therefore itinerant patients or those with no telephone will be excluded from participation in the trial. Note: At recruitment, additional phone contact numbers will be sought from the patient, this may include family members or neighbours, to minimise loss to follow-up

6. Patients presenting to the ED outside of the recruitment hours (Mon-Fri 8 hours per day)

A record will be kept detailing the reason for each exclusion. Demographic details (age, gender, residential location – community or residential care, triage category) will also be collected to identify if the excluded population is different from the sample population to a level reaching statistical significance.

### Data collection tools

#### *Site visit*

The site visit tool will be designed to focus on environmental factors relating to clinical care, and structural processes (such as policies and procedures, training and staff allocation). The survey strategy will assess physical layout, equipment, staffing levels and policies and procedures pertinent to the ED management of geriatric patients.

Each site visit will take two days and involve discussions with a range of staff attached to the ED. This will include: emergency staff specialists; nursing unit managers; pharmacists; allied health and other members from the multidisciplinary team; quality managers; and ED clerical staff. At the completion of the site visit, the data will be reviewed and a list of incomplete questions will be forwarded to the site coordinator to enable the site to provide additional information that may not have been available at the time of the physical site visit. For each structural QI, data will be sought (if relevant) to support the validation of the QI at the level of policy, protocol, processes implemented and audit (regular review to identify if policy and/or processes are adhered to).

• *Prospective data collection*: Several formal tools will be utilised to collect data from patients while they are in the ED. This will include a tool which provide an overview of the health status of an older person both prior to the onset of the acute episode and during the ED visit (interRAI ED Assessment [36]and selected items from interRAI Acute Care [37,38]). Input from the expert panel will be sought prior to finalisation of included tools. Final battery of assessments is anticipated to include the following standardized assessment or screening tools:

• Cognition (Six Item Screener (SIS) [39]; Orientation-Memory-Concentration (OMC) test [40]; RUDAS [41]); interRAI Cognitive Performance Scale(CPS) [42])

• Delirium (Confusion Assessment Method (CAM) [43]; interRAI delirium screener [44])

• Pressure ulcers (The Waterlow Scale [45])

• Pain (0–10 Numeric Rating (Pain Intensity) Scale (NRS) [46-48], Pain Assessment in Advanced Dementia Scale (PAINAD) [49]

• Falls risk (Falls Risk for Older People in the Community (FROP-COM) Screening Tool [50])

• Discharge risk (Identification of Seniors at Risk Tool (ISAR) [22,51,52], Triage Risk Stratification Tool (TRST) [17,53,54], InterRAI risk screeners [55], Rowland [56])

Any additional data items (such as medication data, demographic data) will be added to the data collection tools as required.

#### *Phone follow-up*

A phone follow up data collection form will be created to collect data on:

• Adverse events following the ED episode

• Additional ED or acute care hospital admissions

• Time spent in residential care (respite or newly admitted as permanent resident)

• Pain and medication management following the ED episode

• Patient satisfaction

• Patient perception of clinical decision making and privacy

Phone follow up will occur at 7 and 28 days but data will be combined to provide a summary of the total 28-day period. Two phone calls are scheduled to ensure continuity of contact with the patient and to assist in more reliable recall of information over shorter periods of time.

#### *Emergency department information system (EDIS) data extraction*

A data collection form will be created to identify key information stored on the system relevant to the patient and to compare this with data collection from the patient. The EDIS data will also be used to provide general demographic data relating to patients both included and excluded (gender, age, triage category, residential setting).

#### *Patient medical record*

This record will vary between institutions, being either electronic, paper based or a combination of both. A chart review tool will be designed which focuses on abstraction of data, and minimises the need for ‘interpretation’ of data during the audit process. Where possible, existing chart abstraction tools will be utilized. The final chart review tool will undergo preliminary pilot testing.

#### *Data custodian information on ED episodes and acute care admissions*

Data on the index ED episode, subsequent acute care admission and any additional hospital interactions in the 28 days post ED departure will be sought from the data custodian in each State. International Classification of Diseases (10^th^ revision) [57] codes, primary diagnoses, length of stay and classification of care for each episode of care during the study time period will be requested.

Each data collection set (comprises all the data collection sheets for each phase of data collection) will be matched against the original data matrix to ensure that all required variables are being collected. A database for data entry will be created. Each variable item, coded to match the data collection sheet and carrying a unique variable name, will be recorded in the manual alongside each QI in preparation for analysis of the data set.

### Research staff

A registered nurse with geriatric assessment expertise(site nurse) will be employed for prospective data collection, including the phone follow-up, at each site. All staff will attend the Centre for Research in Geriatric Medicine (CRGM) for comprehensive training prior to data collection commencing. Each enrolled patient will be assessed by the trained research nurse utilising the compilation of data collection tools – this will allow for a comparison of data collected by the research nurse, who will complete chart reviews, and the site nurses, thus allowing an evaluation of the reliability of QI information obtained by chart audit (through triangulation of data). The patient will otherwise undergo usual ED assessment and management.

Two research staff, with nursing backgrounds will be trained to complete the site visits. One site visit will be completed jointly, but scored separately to test the data collection tool with respect to inter-rater reliability. All remaining site visits will be visited by one of the two research staff.

Research nursing staff will be trained to complete the chart review. The data will be collected in a retrospective fashion by trained chart/database abstractors using a standardized chart abstraction protocol – these abstractors will be blinded to the site nurse assessment. The training will include the protocol, supervised practice charts and independent chart review followed by comparison with trainer review. 5% of charts will be co-reviewed to ensure a kappa of > 0.7, which by convention suggests excellent inter-rater reliability [58].

Staff carrying out the data collection will be blinded to the individual QIs. All data items, regardless of the data collection method (prospective, chart review, site visit) will be standalone items and not be grouped or identified in the data collection sheet as linked to an individual QI.

### Data collection

The research nurse at each site will identify eligible patients at the beginning of each shift using the EDIS. All eligible patients will be approached in consecutive order. If a patient becomes ineligible or is excluded, general demographic information will be recorded, along with the reason for ineligibility. For eligible patients, the research nurse will explain the purpose of the study, the range of questions that will be asked and the anticipated duration of the patient’s involvement and seek written consent from the patient or a nominated secondary decision maker for participation.

The research nurse will confirm general contact and demographic information with the patient. The initial data collection questions will focus on the patient’s current condition or situation, and include items relating to cognition, delirium, pain, medications, skin integrity and continence (these questions relate to aspects of health that may change before and during the ED episode). A second series of questions will be related to the patient’s situation prior to the onset of the acute medical condition, the reason for attending the ED, and arrangements for additional care following the ED episode (capacity to get home, additional nursing care, etc.).

If the patient is in the ED for 3 or more hours, the research nurse will return to the patient and repeat the section of the initial data collection questions which focused on the patient’s condition (cognition, delirium, etc.).

Following the patient’s departure from the ED, the research nurse will identify the discharge medications, discharge location, length of stay in the Emergency Department and other general details.

Seven days following the departure from the ED, the research nurse will contact the patient and complete the phone follow up. At 28 days, the phone follow up will be repeated with a small section of repeated questions, which relate to any adverse events in the preceding days.

The site visit is organised separately from the prospective data collection and will occur throughout the data collection period.

The chart reviews will be completed following the end of the prospective data collection period. They will commence no sooner than two months after data collection had been completed. This will enable all relevant information to be filed in the chart. All patient medical records will be recalled and the chart abstractors will review each chart using a pilot tested audit tool.

Finally, no sooner than six months after the end of the prospective data collection, the data custodian will be contacted to request the relevant information regarding the index ED episode and any other hospital events up to and including 28 days post ED departure. The time lapse is to ensure that all data has been received from the State’s hospitals.

### Data compilation

A recruitment database will be completed by the site nurse and forwarded, at regular intervals, to CRGM for review. The recruitment database will hold the general demographic information and the unique research identifies for each consenting patient.

All data, assembled by the research nurse for prospective data collection, will be de-identified and forwarded to CRGM for data entry into an electronic database. Each file will be reviewed by one researcher for completeness prior to data entry. Any issues will be reconciled by request to the research nurse at each site.

A second researcher will review all site visit data and request any missing information from each site coordinator. The data will then be entered into an electronic database ready for analysis.

Finally, the chart abstraction data collection tools will be forwarded to CRGM, identified only by the unique research ID, reviewed by one researcher for completeness and entered into an electronic database.

A separate database will be established for each site. When all data is entered, and checked, the databases will be combined to establish one complete database ready for analysis.

## Phase 3: Expert panel and voting rounds

### Objective

The purpose of the final expert panel is to enable panel to review the preliminary indicators alongside the data from the field study (phase 2) and revise or exclude indicators prior to the voting round. The voting rounds will culminate in the assembly of a final QI set that will reflect quality of care in terms of structure, process and outcomes.

### Design

This phase will comprise the latter stages of analysis of results of the field study, preparation of reports to inform the expert panel, a two day seminar to consider the findings of the field study and assembly of the final QI set with associated recommendations.

A formal report will be prepared for general scrutiny in addition to publication for the peer-reviewed literature. A formal procedure for selection of the final QI set will follow the expert panel deliberations, similar to that used in assembly of the Assessing Care Of Vulnerable Elders (ACOVE) indicators [59]. This process involves two rounds of anonymous ratings on a risk-benefit scale with a teleconference group discussion occurring between rounds [60,61].

### Data analysis

Primary analysis will be to evaluate the new QIs. The QIs will be adjusted for ascertainment and selection bias through risk adjustment procedures [58]. The determination of appropriate case-mix and risk adjustment procedures will involve simple bi-variable descriptive statistics (correlations, mean differences). Good candidates for adjustment will be included as matching criteria in the QI adjustment process. The QI adjustment method will use a procedure that has the advantage of being quasi-parametric, involving matching individual patients in target EDs to randomly selected patients from other EDs. This counterfactual contrast will include a re-sampling procedure and allow QIs to be expressed as odds ratios or expected proportions given an overall average rate and an empirically based replication (i.e. confidence) interval. Relative to extant methods of risk adjustment this approach is relatively simple, can be implemented in clinical populations of small size and represents as perfect as possible adjustment for differences in patient mix across clinical settings.

The reliability of QI scores will be evaluated by multiple bootstrapped split-half correlations of patient samples and time-to-time correlations of repeated QI scores. This is a unit-level analysis, where for each ED we will use a bootstrapping data augmentation approach to generate 20 random half samples of patients.

Consideration of the issues specific to patients with cognitive impairment, nursing home residents and those patients requiring palliative care will result in an additional analysis of QI data to identify whether any QIs are specifically significant for these sub-groups.

Comparisons with SAEM QIs will use standard methods for comparing correlation coefficients for the contrasting reliability coefficients, and cross tabulations of tertiles of QIs in similar domains for the validity assessment.

### Voting

Following the final expert panel, the indicators will be presented to the expert panel in a summary document. In the document, each indicator will be described in relation to the agreed name, denominator, numerator and exclusion criteria. A short summary of relevant evidence supporting the indicator and a précis of the panels’ discussion in relation to how well the indicator aligned with the selection criteria, will be included. There will be graphical representation of the field study data, including prevalence (raw scores) of the trigger rates, and percentage scores.

There will be a formal voting process, involving two voting rounds, following the RAND-UCLA Appropriateness Method [62,63]. The panel will be asked to rate each indicator with a score from one to nine based on its validity when considered in relation to the selection criteria. The selection criteria include:

• Criteria 1: **Quality of Care indicator** - Adequate scientific evidence *or* professional consensus supported a link between the process specified by the indicator and a health benefit to the patient; an ED with high rates of adherence to the indicator would be considered a higher-quality provider

• Criteria 2: **Measurement accuracy** - Ideally the indicator would be measured using a gold standard measure or a measure with proven robust attributes for the measured population when administered appropriately; the indicator measures what it is meant to measure

• Criteria 3: **Provider Control** - An ED influences a majority of the factors that determine the outcome of the indicator (relevant to the inpatient episode of care)

• Criteria 4: **Generalisability** – The indicator is relevant to a high proportion of the targeted population

• Criteria 5: **Responsiveness** - The indicator is responsive to changes over time; that is, it will be possible to identify and measure the impact of interventions designed to improve care. (i.e. evidence that there are interventions which can lead to improvement in care)

• Criteria 6: **Event Rate** - Occurs frequently and is of sufficient significance that monitoring should occur

Voting sheets will be returned to CRGM, where they will be collated. A second round of voting sheets will be distributed to the panel. Each individualised voting sheet will include: the de-identified votes of the panel (i.e. how many panel members voted ‘1’, how many voted ‘2’, etc.) for each indicator; the actual vote of the panel member from round one; summary of the panel votes including the median vote; the mean standard deviation from the median; presence of agreement (or disagreement) in relation to that indicator; result of the round one vote (indicator valid, undecided or invalid). Panel summary statistics will be calculated after removing the highest and lowest vote for each indicator (i.e. the most extreme votes).

Agreement is decided by calculating the Interpercentile Range Adjusted for Symmetry (IPRAS) and the Interpercentile Range (IPR) [62]. If the IPRAS is larger than the IPR then there is agreement in the panel on a particular indicator. The indicator is valid if the median score is between seven and nine, and the panel are in agreement. A median with a decimal of 0.5 or higher is rounded up.

As this voting process is a consensus method, there will be a teleconference to discuss the voting round. The focus of discussion is on indicators where there was disagreement in round one. In some instances, disagreement occurs because of a misunderstanding or lack of clarity in the definition. This discussion allows the opportunity to clarify the definition such that it improves the usefulness of the final indicator. In some instances, the disagreement occurs because of a difference in opinion. Given the multi-disciplinary nature of the panel, this teleconference enables one last opportunity for evidence to be highlighted in support of a point of view.

The panel then vote for a second time on all indicators. They can repeat their vote or move their vote up or down the scale to strengthen the impact of their opinion. All indicators identified as valid in this second round of voting, will be incorporated in the final set. If there is one care domain where no valid indicators are identified, but there are indicators where the vote is ‘undecided’ (median score was 4–6 or there was disagreement (IPRAS less than or equal to IPR), then the undecided indicator with the highest median (taking into account decimal places) will be included in the final indicator set.

### Integration of findings

Dissemination of findings will be undertaken by publication in peer reviewed Emergency medicine and Medical Administration Journals of:

1. Scientific reviews of the literature undertaken to allow optimal evidence-base for development of robust QIs

2. A final recommended QI set for care of elderly in the ED

Following the above project, the finalised set of QIs will be subjected to a more widespread validation study. Results of this study will be a validated set of QIs for care of older persons in ED – these will be presented to key Australian and international Emergency Medicine Colleges and Societies and to national and international accrediting boards for consideration of ratification. In addition, presentations are planned at national and international conferences to communicate results to attendees. Finally, the use of these QIs by clinical investigators as outcome measures, supplementary to their project specific measures, will be encouraged by the research team.

Given that existing QIs will be compared to indicators developed in this project, stakeholders will be empowered to choose those indicators that will most optimally fulfil their specific goals.

## Discussion

Quality indicators (QI) are quantitative measures that may be utilised to enable levels of performance to be determined and, as part of a quality management system, provide opportunity for benchmarking and improved care delivery [29]. They may also support accreditation, regulation, and patient and healthcare purchaser choice. This study will result in a suite of QIs for use in the ED care of elderly that will be:

1. Valid

2. Derived utilizing clinical data items from multiple sources, including a site audit, patient interview, administrative databases, the interRAI ED assessment tool and the medical record

3. Feasible in terms of both cost & measurement

4. Assess the full spectrum of Donabedian’s domains including structure, process and outcomes [64]

5. Designed utilizing data items and processes that are not unique to any one particular developed nation.

The predicted burgeoning in the number of older persons presenting to EDs combined with the recognised quality deficiencies in ED care delivery to this population, highlight the need for a quality framework for the care of older persons in ED. Additionally, high quality of care is associated with improved survival & health outcomes of elderly patients [28]. The development of well-selected, validated and economical QIs will allow appropriate targeting of resources (financial, education or quality management) to improve quality in areas with maximum potential for improvement. Conversely, the “blind” application of QIs not designed for nor tested in the ED setting, particularly in the absence of appropriate risk adjustment, may result in inappropriate misdirection of funding.

## Abbreviations

ED: Emergency department; QI(s): Quality indicator(s).

## Competing interests

The authors declare that they have no competing interests. Authors Gray and Jones are Fellows, and Martin-Khan is an Associate Fellow, of the interRAI research consortium, which is a not-for-profit organization registered in the United States. Fellows contribute to the interRAI effort on a purely voluntary basis.

## Authors’ contributions

EB and LG established the project and the project team. MMK conceptualised the research design. MMK, EB, LS, and LG jointly refined the research methodology and wrote the research protocol. MMK, EB and LS jointly drafted the first manuscript and extensively revised following feedback from other authors. LG and RJ extensively reviewed the manuscript and contributed to the revisions prior to submission. MMK coordinated the submission process. All authors read and approved the final manuscript.

## Pre-publication history

The pre-publication history for this paper can be accessed here:

http://www.biomedcentral.com/1471-227X/13/23/prepub
